# Supplementary figures and images for: Clostridioides difficile toxins alter host metabolic pathway and bile acid homeostasis gene expression in colonic epithelium
Source: Infect Immun. 2025 Jun 30;93(8):e00150-25. doi: 10.1128/iai.00150-25 (PMC12341375; doi:10.1128/iai.00150-25)

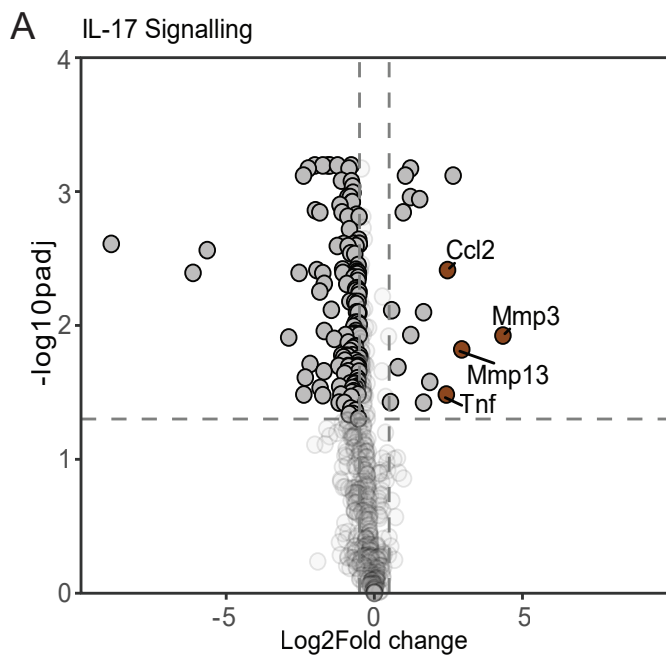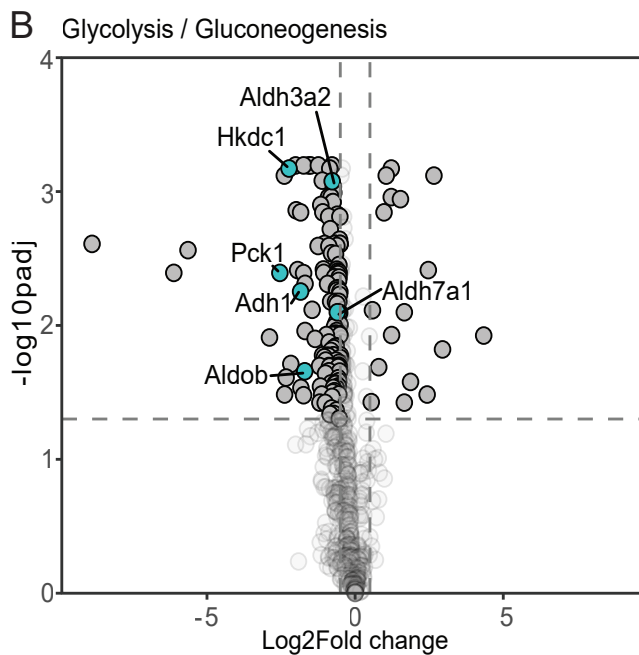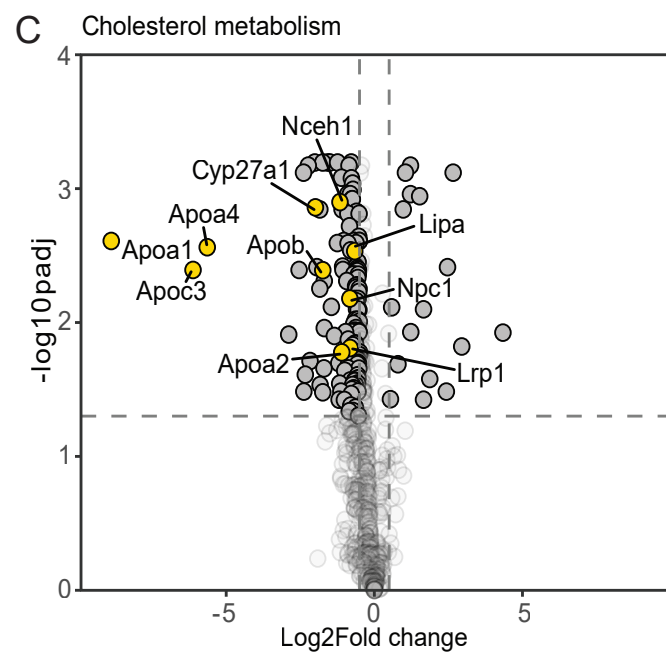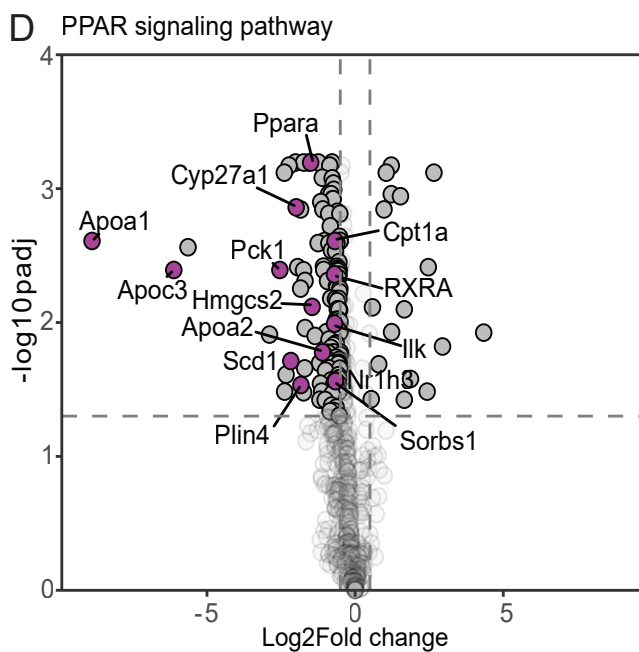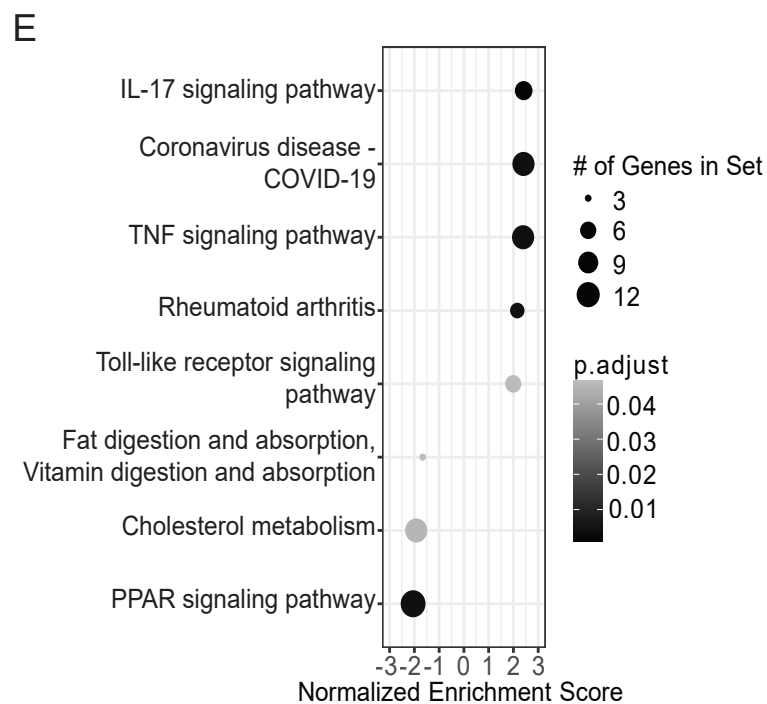

Supplement: Fig. S1 — Individual volcano plots of genes that changed in expression during C. difficile infection compared to antibiotic treated mice. [file iai.00150-25-s0001.pdf]

Caco-2 cells

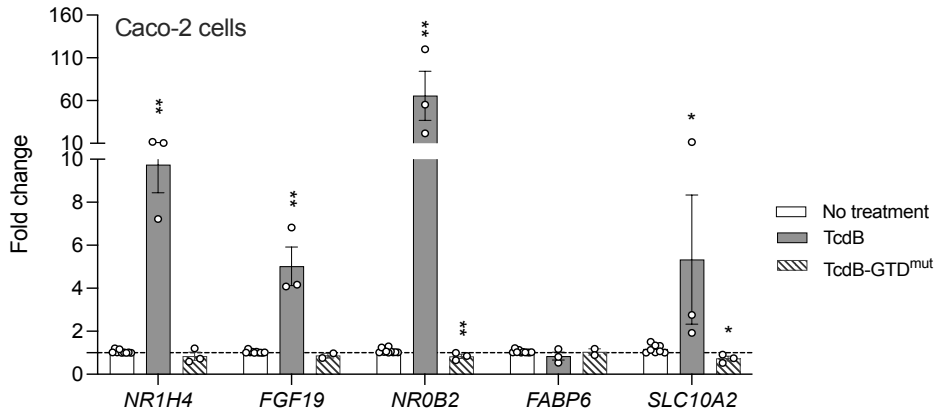

Supplement: Fig. S2 — TcdB alters FXR regulatory gene expression by disrupting GTPase signaling. [file iai.00150-25-s0002.pdf]
