# Supplementary material for: Clostridioides difficile toxins alter host metabolic pathway and bile acid homeostasis gene expression in colonic epithelium
Source: Infect Immun. 2025 Jun 30;93(8):e00150-25. doi: 10.1128/iai.00150-25 (PMC12341375; doi:10.1128/iai.00150-25)
Supplement: Supplemental material — All Supplemental figure legends. [file iai.00150-25-s0003.docx]

**Supplemental material:**

**Supplemental Figure 1: Individual volcano plots of genes that changed in expression during *C. difficile* infection compared to antibiotic treated mice**. Volcano plots of genes differentially expressed in CDI mice compared to Cef mice. KEGG pathways are labeled in A) IL-17 signaling, B) Glycolosis/Gluconeogenesis, C) Cholesterol metabolism, and D) PPAR signaling pathway. E) Gene set enrichment analysis (GSEA) identifying gene clusters that changed the most in CDI compared to Cef mice.

**Supplemental Figure 2: TcdB alters FXR regulatory gene expression by disrupting GTPase signaling.** Caco-2 wells were treated with 100 pM of either TcdB, or 100 pM of TcdB-GTP^mut^ and incubated for 24 hr. NT, no treatment control only contained media with no other additions. Asterisks denote statistical significance determined by Mann-Whitney t-test compared to the No treatment control, ***p* < 0.01.

**Supplemental Table 1**: Additional genes used in NanoString analysis

**Supplemental Table 2**: Probes used in NanoString analysis
