# Supplementary material for: Clostridioides difficile toxins alter host metabolic pathway and bile acid homeostasis gene expression in colonic epithelium
Source: Infect Immun. 2025 Jun 30;93(8):e00150-25. doi: 10.1128/iai.00150-25 (PMC12341375; doi:10.1128/iai.00150-25)
Supplement: Table S1 — Additional genes used in NanoString analysis. [file iai.00150-25-s0004.pdf]

Supplemental Table 1: Additional genes used in NanoString analysis

Gene Name	Protein Name	Significant Differential Expression in cecum (Log$_2$ fold change)
**Nuclear receptors**		
Nr1i1	VDR	Yes (-1.03)
Nr1i2	PXR	No
Nr1i3	CAR	Yes (-1.94)
Rxra	RXRA	Yes (-0.697)
Gpbar1	TGF5	Not detected
Nr1h2	LXRb	Yes (-0.528)
Nr1h3	LXRa	Yes (-0.672)
Nr1h4	FXR	No
Nr0b2	SHP	Not detected
**Intestinal BA transport**		
Slc10a2	ASBT	No
Slc51a	OSTa	Yes (-1.69)
Slc51b	OSTb	No
Fabp6	IBABP, FABP6	No
Abcc2	ABCC2	Not detected
Slc9a3	NHE3	No
**Enterohepatic BA signaling**		
Fgf15	FGF15	Not detected
**Liver BA transport**		
Baat	BAAT	Not detected
Slc10a1	NTCP	Not detected
**Liver BA transmembrane receptors**		
Klb	b-Klotho	Not detected
Fgfr4	FGFR4	No
Vldlr	VLDLR	No
ldlr	LDLR	No
**BA synthesis**		
Cyp27a1	CYP27a1	Yes (-1.99)
Cyp7a1	CYP7a1	Not detected
Cyp7b1	CYP7b1	No
Cyp8b1	CYP8b1	No
